# SARS-CoV-2 Variant Pathogenesis Following Primary Infection and Reinfection in Syrian Hamsters

**DOI:** 10.1128/mbio.00078-23

**Published:** 2023-04-10

**Authors:** Jessica Plunkard, Kathleen Mulka, Ruifeng Zhou, Patrick Tarwater, William Zhong, Margaret Lowman, Amanda Wong, Andrew Pekosz, Jason Villano

**Affiliations:** a Department of Molecular and Comparative Pathobiology, Johns Hopkins University, Baltimore, Maryland, USA; b Research Animal Resources, Johns Hopkins University, Baltimore, Maryland, USA; c W. Harry Feinstone Department of Molecular Microbiology and Immunology, The Johns Hopkins Bloomberg School of Public Health, Baltimore, Maryland, USA; d Department of Epidemiology and Biostatistics, Texas A&M University, College Station, Texas, USA; Texas Biomedical Research Institute; University of Pennsylvania

**Keywords:** breakthrough infection, COVID-19, SARS-CoV-2, Syrian hamster, variant, animal models, coronavirus, laboratory animals

## Abstract

Severe acute respiratory syndrome coronavirus 2 (SARS-CoV-2), the causative agent of COVID-19, has evolved into multiple variants. Animal models are important to understand variant pathogenesis, particularly for variants with mutations that have significant phenotypic or epidemiological effects. Here, cohorts of naive or previously infected Syrian hamsters (Mesocricetus auratus) were infected with variants to investigate viral pathogenesis and disease protection. Naive hamsters infected with SARS-CoV-2 variants had consistent clinical outcomes, tissue viral titers, and pathology, while hamsters that recovered from initial infection and were reinfected demonstrated less severe clinical disease and lung pathology than their naive counterparts. Males had more frequent clinical signs than females in most variant groups, but few sex variations in tissue viral titers and lung pathology were observed. These findings support the use of Syrian hamsters as a SARS-CoV-2 model and highlight the importance of considering sex differences when using this species.

## INTRODUCTION

Severe acute respiratory syndrome coronavirus 2 (SARS-CoV-2) variants are genetic lineages of the virus with mutations that alter virus replication, disease, transmission, or evasion of adaptive immune responses (1). These were initially noted in early 2020, only a few months after the first reported COVID-19 cases (2). Multiple genetically distinct variant lineages have since emerged, and have been classified based on their genomic sequences via the Pango dynamic nomenclature system (3). An epidemiological classification system was also established to characterize variants with potentially deleterious human health consequences into three categories: variants under monitoring (VUM), variants of interest (VOI), and variants of concern (VOC), with VOC having the most potential for impacting public health and disease outcomes (2). The WHO further labels individual VOC and VOI using the Greek alphabet (2). Table 1 identifies the isolate, Pango lineage, and WHO Greek alphabet designation (as applicable) for variants used in this study.

**TABLE 1:** List of variants utilized

Variant full name	Collection date	Pango lineage	WHO name (as applicable)
SARS-CoV-2/USA-WA1/2020	19 January 2020	A.1	
SARS-CoV-2/USA/MD-HP02153/2021	2 February 2021	A.2.5	
SARS-CoV-2/USA/MD-HP00076/2020	11 March 2020	A.3	
SARS-CoV-2/USA/MD-HP12112/2021	1 January 2021	B.1.1.207	
SARS-CoV-2/USA/MD-HP01542/2021	21 January 2021	B.1.351	Beta
SARS-CoV-2/ USA/CA-Stanford-02_S43/2021	13 January 2021	B.1.427	Epsilon
SARS-CoV-2/USA/MD-HP03867/2021	1 April 2021	P.1.17	Gamma
SARS-CoV-2/USA/MD-HP05660/2021	2 May 2021	AY.106	Delta
SARS-CoV-2/USA/MD-HP20874-PIDUYWZOWA/2021	27 November 2021	BA.1.18	Omicron
SARS-CoV-2/USA/MD-HP06587-PIDGNNWCBG/2021	15 June 2021	B.1.621	Mu

Animal models have been used to study SARS-CoV-2 pathogenesis, transmission, vaccine candidates, and therapeutics (4). As with previous coronaviruses, Syrian hamsters stand out as an animal model (5). The virus replicates efficiently in their airways and nasal passages without progressing to severe disease in adult animals (6–8), modeling the outcome most seen in human patients. Syrian hamsters offer a valuable resource for understanding the growing complexities of COVID-19, such as the effect of multiple infections on morbidity and long-term sequalae (9–11), the protective immunity conferred from vaccines and previous infections (12–15), and the impact variant differences have on these outcomes (16–20). Here, differences in the pathogenesis of SARS-CoV-2 variants in naive or previously infected hamsters were investigated using clinical outcomes, lung histopathology, and respiratory tissue viral titers. We hypothesized that hamsters would display mild phenotypes following variant infection, similar to previous reports, and that reinfected hamsters would have reduced clinical signs and milder lung histopathology across variant groups.

## RESULTS

**FIG 1:**
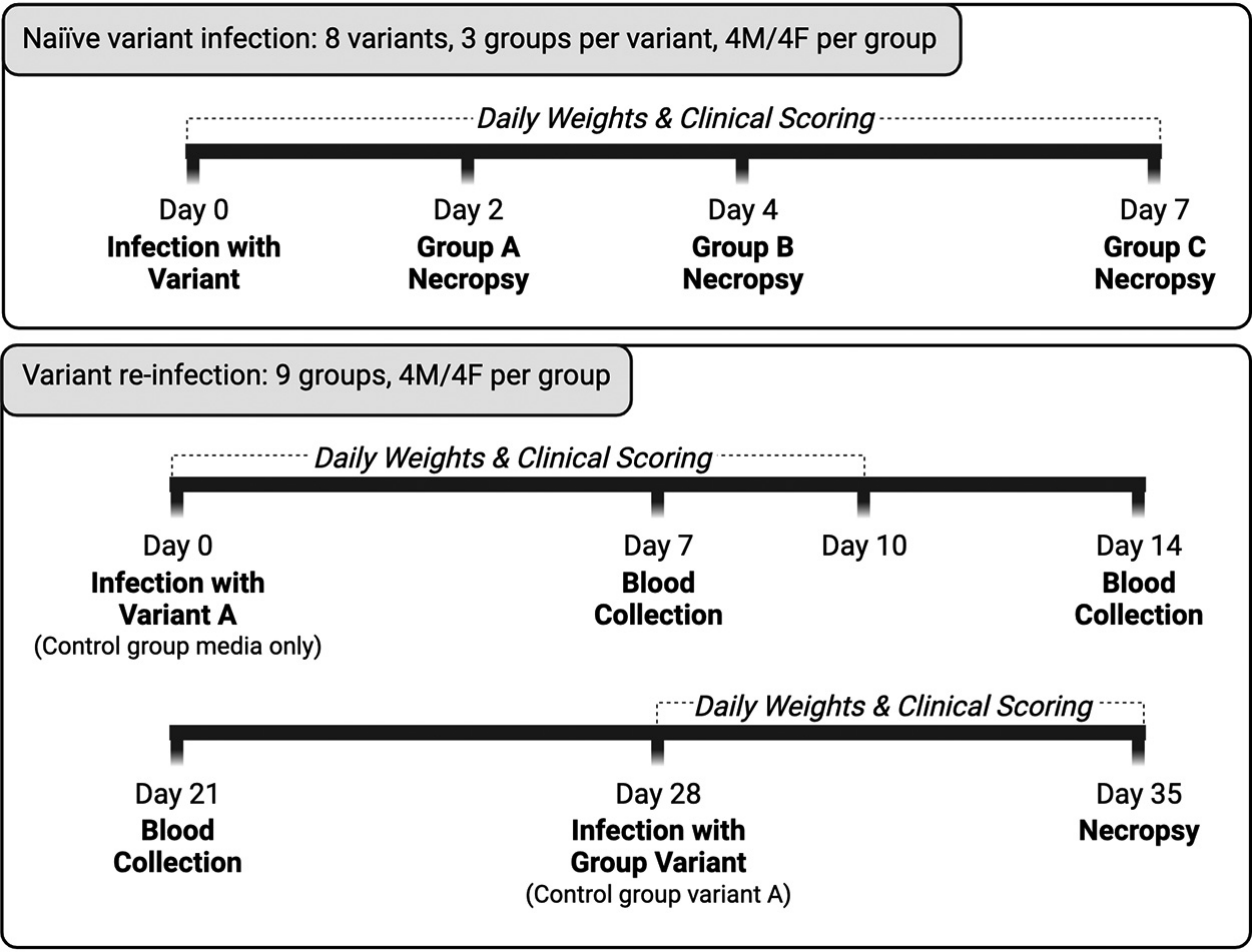
Experimental timeline. (A) Timeline for the naive variant infection groups. Hamsters were inoculated with a variant and then clinically scored and weighed daily until euthanasia at 2, 4, or 7 dpi. (B) Variant reinfection groups’ timeline. Hamsters were inoculated with variant A and then received clinical scoring and weighed daily until 10 dpi. At 28 dpi, hamsters were reinfected with a variant, followed by an additional 7 days of weighing and clinical scoring before euthanasia at 35 dpi. Blood was collected at 7, 14, and 21 dpi.

Naive and previously infected hamsters were infected with SARS-CoV-2 variants and underwent clinical monitoring, histopathologic lung scoring, and respiratory tissue viral titer analyses. Figure 1A shows the sample collection timeline for each of these experimental designs.

### Naive infection with SARS-CoV-2 variants.

**FIG 2:**
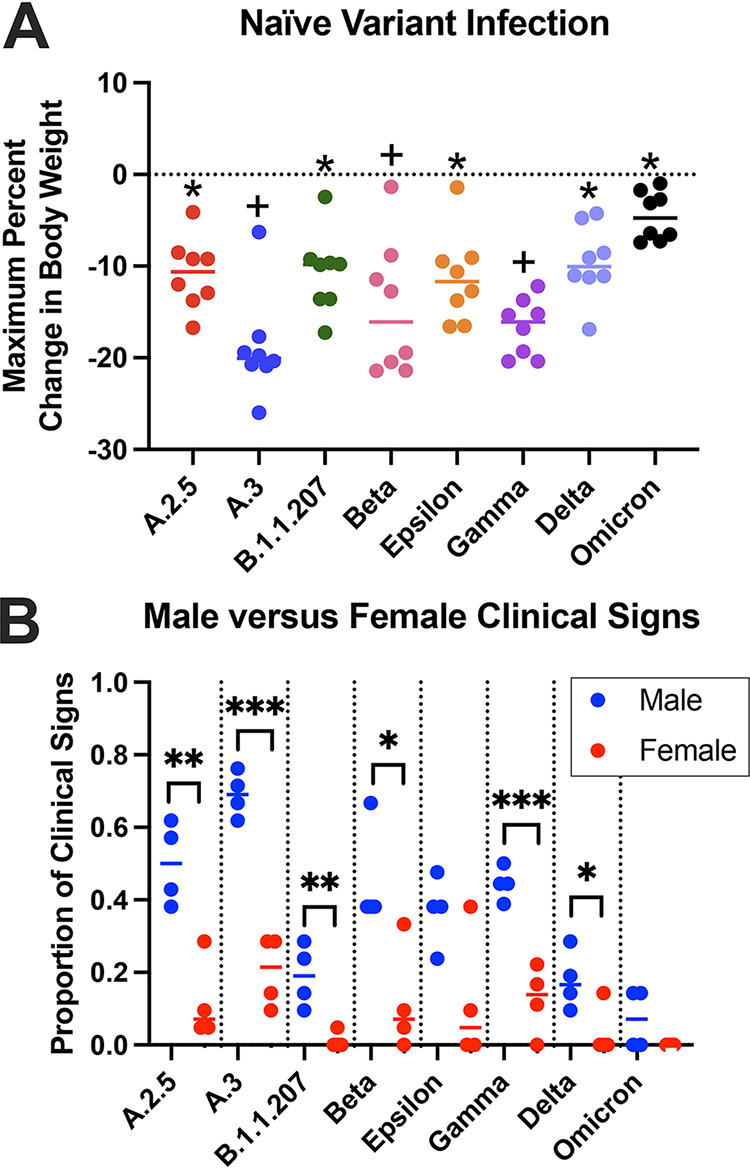
Clinical signs following naive infection. (A) Maximum percentage of body weight change over 7 dpi. Using a one-way ANOVA, few significant differences in body weight changes were observed between variant groups, apart from the Omicron group experiencing significantly less weight loss than several groups (denoted with +) and A.3 group experiencing significantly more weight loss than several other groups (denoted with *). (B) Although few sex differences were observed between variant groups for body weight change, significant differences were noted in the frequency of other clinical signs between all groups except Omicron and Epsilon (*, *P* < 0.05; **, *P* < 0.01; ***, *P* < 0.001).

**(i) Clinical outcomes.** Clinical outcomes were monitored daily to evaluate differences between variants and sexes. Hamsters lost body weight (BW) over the first 6 days postinfection (dpi) and began regaining weight at 7 dpi, with some differences in mean BW appreciated. Groups infected with A.3 exhibited significantly more BW loss than groups infected with A.2.5, B.1.1.207, Epsilon, Delta, and Omicron, losing up to 26% BW by 6 dpi. Omicron-infected animals lost significantly less BW than the A.3-, Beta-, and Gamma-infected groups, with a maximal loss of only 6% BW (Fig. 2A). There was no significant sex-associated impact on BW.

Other clinical signs were recorded as present or absent after observation in the home cage before handling. Hamsters exhibited rough hair coat, orbital tightening, and hunched posture. Males displayed these more frequently than females in all groups, with six out of eight groups showing a significant sex difference (Fig. 2B). When observed over 7 dpi, males infected with Delta and Omicron had clinical signs less frequently than males infected with A.2.5, A.3, Beta, and Gamma. The Omicron-infected males also had less frequent clinical signs than Epsilon-infected males. Female hamsters exhibited no significant differences in clinical signs between variant groups.

**FIG 3:**
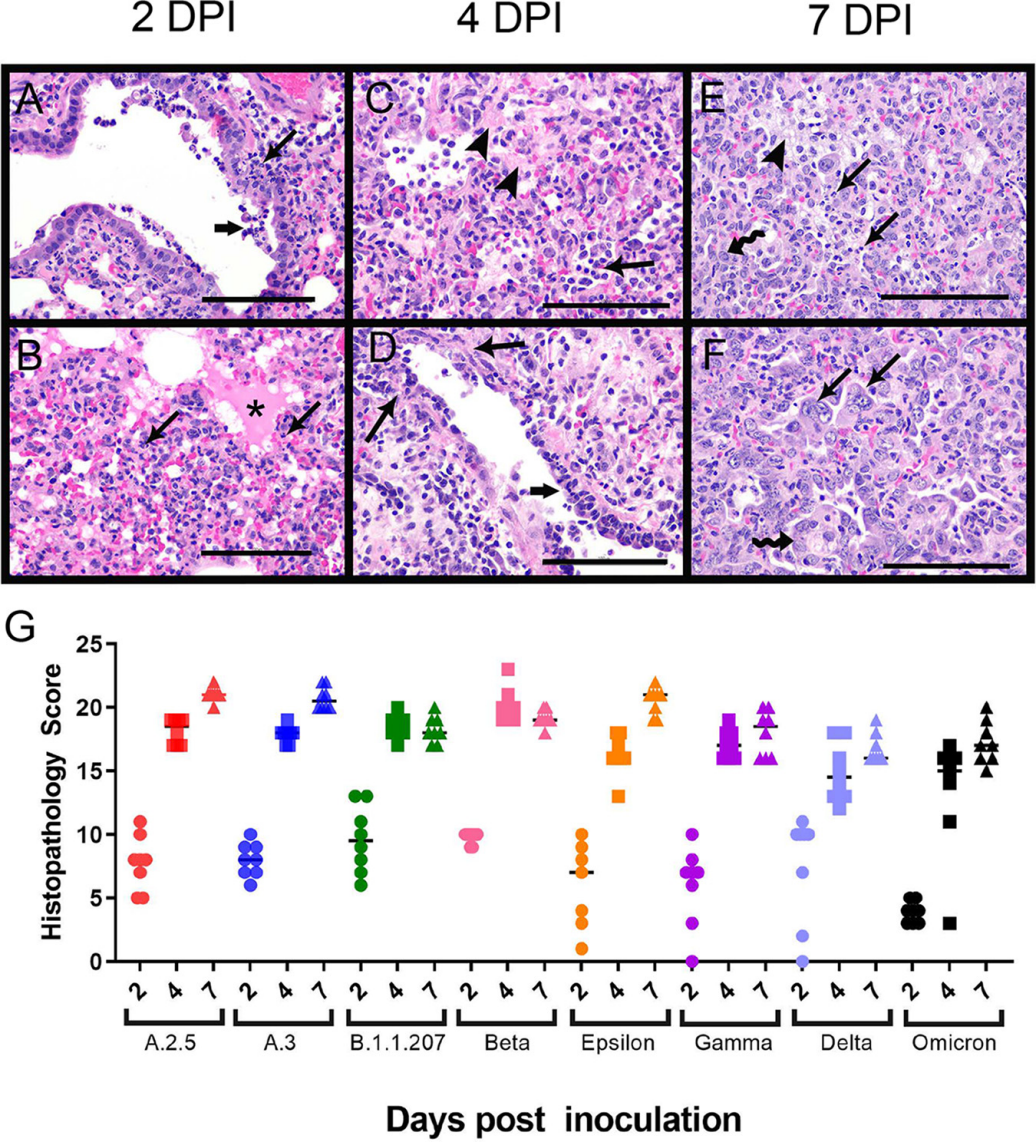
Histopathology of the lungs following naive infection. Histopathologic changes of the lungs were consistent between variants at each time point. Representative images from each time point from different variants are shown. (A and B) Two days postinfection. (A) Suppurative bronchitis and bronchiolitis (long arrow) and bronchial epithelial cell necrosis along with inflammatory cell and necrotic cellular debris within the airways (short arrow) as observed in the Epsilon variant; (B) alveolar edema (*) and inflammatory cells (arrows), including large numbers of neutrophils and macrophages as observed in Gamma variant. (C and D) Four days postinfection. (C) Organizing fibrin in the alveoli (arrowhead) and numerous inflammatory cells, including neutrophils and macrophages (arrow) as observed in the B.1.1.207 variant; (D) vasculitis/vascular endothelialitis characterized by loss of vessel wall integrity and eosinophilic proteinaceous material within the vessel wall (long arrows) and subendothelial mononuclear cells and neutrophils with endothelial cell swelling and necrosis (short arrow) as observed in the B.1.1.207 variant. (E and F) Seven days postinfection. (E) Organizing fibrin within the alveoli (arrowhead), degenerate inflammatory cells within alveoli (arrows), and type II pneumocyte hyperplasia (squiggle arrow) as observed in the A.2.5 variant; (F) multinucleated sloughed epithelial cells (arrows), type II pneumocyte hyperplasia (squiggle arrow), and intra-alveolar inflammatory cells as observed in the A.3 variant. Scale bars represent 100 μm. (G) Histopathology scores of naive infection groups. Please see Fig. S2 for a complete list of *P* values from multiple comparisons and one-way ANOVA. Each variant exhibited a similar trend with respect to scores at 2 dpi (circles), 4 dpi (squares), and 7 dpi (triangles). Across time points, Omicron-infected animals had lower histopathology scores than animals infected with A.3, B.1.1.207, and Beta variants at 2 dpi, all variants except Epsilon and Delta at 4 dpi, and all variants except B.1.1.207, Gamma, and Delta at 7 dpi. Animals infected with Delta had lower histopathology scores than those infected with B.1.1.207 and Beta variants at 4 dpi and all variants except B.1.1.207, Gamma, and Omicron at 7 dpi.

**(ii) Histopathology.** Histopathological changes were evaluated to monitor respiratory disease progression 2, 4, and 7 dpi. Changes within the lungs were largely consistent across variants, with the majority of lesion variability observed between different days postinfection. At 2 dpi, lung lesions consisted of small to moderate amounts of intraluminal bronchial and bronchiolar inflammatory and epithelial cellular debris, intra-alveolar macrophages, neutrophils, necrotic cellular debris, fibrinous exudate, and hypertrophied vascular endothelial cells (Fig. 3A and B). In most variant infections, there was mild suppurative bronchitis and bronchiolitis, except for A.2.5 and Epsilon, where these features were only present in one or two animals, respectively. Other variable changes across groups include bronchial epithelial hyperplasia, alveolar hemorrhage and edema, and perivascular lymphocytic aggregates. Epithelial syncytia were rare.

10.1128/mbio.00078-23.2 FIG S2 Percentage of weight change over 7 days following variant naive infection and variant reinfection. (A to H) Naive hamsters consistently lost weight after infection, while reinfected hamsters showed little to no initial weight loss followed by weight gain over 7 dpi. Download FIG S2, TIF file, 13.6 MB. Copyright © 2023 Plunkard et al. 2023 Plunkard et al. https://creativecommons.org/licenses/by/4.0/ This content is distributed under the terms of the Creative Commons Attribution 4.0 International license.

At 4 dpi, alveolar and perivascular edema was still appreciable and similar in severity to the edema seen at 2 dpi. However, the perivascular lymphocytic aggregates, intra-alveolar macrophages, neutrophils, necrotic cellular debris, and organized fibrinous exudate were more severe at 4 dpi than at 2 dpi. Additional lesions at 4 dpi included atypical type II pneumocyte hyperplasia and vasculitis (Fig. 3C and D). Vasculitis, when observed, was usually present in medium-size arteries and veins and was characterized by loss of vessel wall integrity due to transmural effacement by inflammatory cells and necrosis, eosinophilic proteinaceous material within the vessel wall, or subendothelial mononuclear cells and neutrophils with endothelial cell swelling and necrosis. Similar lesions in human patients are described as endothelialitis (21), which has also been used in the literature on hamster models (22–25). Suppurative bronchitis and bronchiolitis were variably present across groups at 4 dpi. Most animals infected with A.2.5, A.3, Epsilon, Gamma, and Delta demonstrated this lesion. However, fewer animals infected with Omicron and Beta variants and no animals infected with B.1.1.207 had this lesion.

At 7 dpi, the most consistent and striking lesion appeared as areas of robust, atypical type II pneumocyte hyperplasia (Fig. 3E and F), which was seen in all animals and was usually present in at least 50% of tissue sections. Cells were up to 40 μm in diameter, cuboidal to polygonal, with round to oval nuclei up to 15 μm in diameter that contained finely stippled chromatin and 1 to 3 prominent nucleoli. These cells had variable amounts of cytoplasm and occasionally were bi- or trinucleate, with numerous mitotic figures. Within the alveolar spaces and expanding the alveolar septa were large numbers of macrophages, with fewer neutrophils and lymphocytes, as well as fibrinous eosinophilic exudate. There was also bronchial and bronchiolar luminal necrotic cellular debris bronchial epithelial hyperplasia, perivascular lymphocytic aggregates, and vascular endothelial hypertrophy.

Lung histopathology was also evaluated using a scoring system shown in Table S1 in the supplemental material. Most variants exhibited a similar histopathological trend, with scores increasing over time and no significant difference between sexes appreciated (Fig. 3G). An exception was animals infected with B.1.1.207 and Beta variants, which had higher scores at 4 dpi than 7 dpi. These animals also had the highest 4 dpi scores across all variant groups. The lower scores at 7 dpi were due to lower percentages of tissue affected, smaller clusters of perivascular lymphocytes, and fewer animals with multinucleated or atypical bronchial epithelial cells. Omicron-infected animals had statistically lower histopathology scores than those infected with A.3, B.1.1.207, and Beta at 2 dpi, all variants except Epsilon and Delta at 4 dpi, and all variants except B.1.1.207, Gamma, and Delta at 7 dpi. Delta-infected animals had lower histopathology scores than B.1.1.207- and Beta-infected animals at 4 dpi and all variants except B.1.1.207, Gamma, and Omicron at 7 dpi. Despite overall lower histopathology scores, Omicron-infected animals demonstrated similar lesions to those associated with other variants (see Fig. S1 in the supplemental material).

10.1128/mbio.00078-23.1 FIG S1 Histopathology of the lungs, Omicron variant. (A and B) Two days postinfection. (A) Suppurative bronchitis (arrowheads), with bronchial epithelial cell degeneration and necrosis; (B) intra-alveolar organized fibrin (arrows) and increased inflammatory cells, including neutrophils and macrophages (arrowhead) within the alveolar septa and spaces. (C and D) Four days postinfection. (C) Vasculitis/vascular endothelialitis characterized by subendothelial aggregates of neutrophils and mononuclear cells (arrows), as well as perivascular accumulation of inflammatory cells, including neutrophils and macrophages (*); (D) organized fibrin (*) within the alveoli along with increased numbers of inflammatory cells, including neutrophils and macrophages (arrowheads). (E and F) Seven days postinfection. (E) Low magnification indicating the extent of the consolidation (*) of the alveoli; (F) higher magnification indicating type II pneumocyte hyperplasia (arrow), inflammatory cells, including neutrophils (arrowhead), macrophages, and fewer lymphocytes, and multinucleated epithelial cells (squiggle arrow). The scale bar represents 100 μm in all images. Download FIG S1, TIF file, 6.9 MB. Copyright © 2023 Plunkard et al. 2023 Plunkard et al. https://creativecommons.org/licenses/by/4.0/ This content is distributed under the terms of the Creative Commons Attribution 4.0 International license.

10.1128/mbio.00078-23.4 TABLE S1 Histopathologic scoring system used blind by a board-certified veterinary pathologist to analyze hamster lung tissue. Download Table S1, DOCX file, 0.03 MB. Copyright © 2023 Plunkard et al. 2023 Plunkard et al. https://creativecommons.org/licenses/by/4.0/ This content is distributed under the terms of the Creative Commons Attribution 4.0 International license.

**FIG 4:**
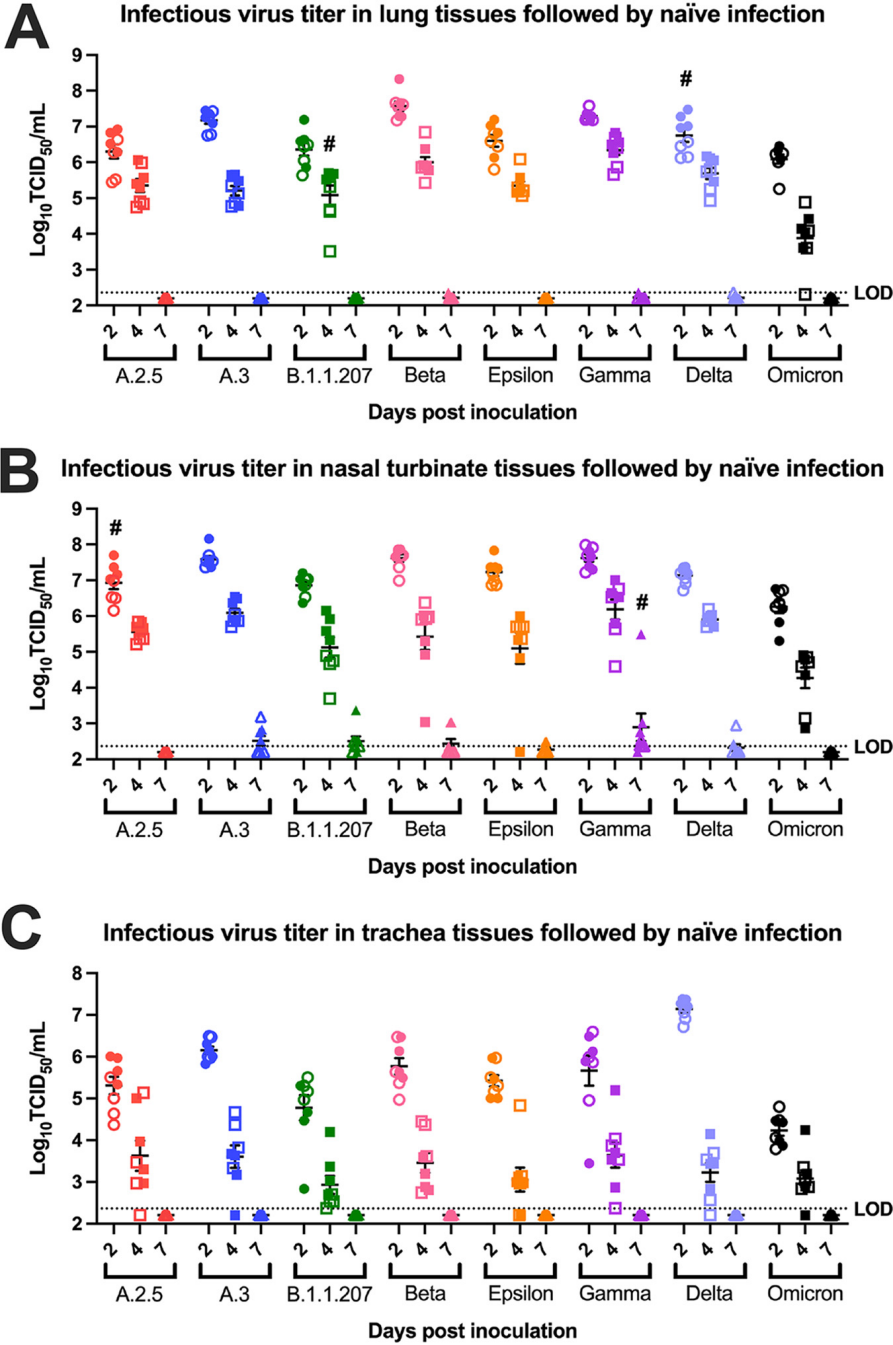
Viral titers in respiratory tissues following naive infection. (A) Mean infectious viral titers in lung tissues at 2 dpi (circles), 4 dpi (squares), and 7 dpi (triangles) after naive infection (*n* = 8). Significant differences in tissue viral titers were observed between some variants at 2 dpi and 4 dpi. Notably Beta and Gamma had higher titers and Omicron had lower titers at both 2 dpi and 4 dpi. No significant difference was observed at 7 dpi. Groups that displayed sex differences in viral titers are indicated by #, with males being higher than females. Hollow circles, squares, and triangles represent data points for female animals. (B) Mean infectious viral titers in nasal turbinate tissues at 2 dpi (circles), 4 dpi (squares), and 7 dpi (triangles) after naive infection (*n* = 8). Few significant differences in viral titers were observed between variants, apart from Omicron having lower titers at 2 dpi and 4 dpi. No significant difference was observed at 7 dpi. Groups that displayed sex differences in viral titers are indicated by #, with males being higher than females. Hollow circles, squares, and triangles represent data points for female animals. (C) Mean infectious viral titers in trachea tissues at 2 dpi (circles), 4 dpi (squares), and 7 dpi (triangles) after naive infection (*n* = 8). Delta had the highest titers in all variants tested, and Omicron had lower titers than all other variants at 2 dpi. No significant differences in tracheal titers were observed between variants at 4 dpi or 7 dpi or between sexes at all time points. Hollow circles, squares, and triangles represent data points for female animals. Viral titer was measured by TCID_50_ assay, with a limit of detection at 2.36 log_10_ TCID_50_s/mL. One-way ANOVA was done on all three tissue types with Turkey’s multiple-comparison tests (*P* < 0.05). Please see Table S4 for the complete list of *P* values for multiple comparisons and one-way ANOVA.

**(iii) Viral titers.** Lung, tracheal, and nasal turbinate titers were measured to evaluate viral tropism in respiratory tissues over time. Overall, the highest viral titers were observed at 2 dpi in all tissue types, with lungs and nasal turbinates having higher titers than the trachea (Fig. 4A to C). All variant groups displayed a quick decrease in infectious virus load at 4 dpi, while no infectious virus was detected in the lung or tracheal samples at 7 dpi (Fig. 4A to C).

10.1128/mbio.00078-23.7 TABLE S4 Results of multiple comparisons and one-way ANOVA of viral titer at 2, 4, and 7 dpi for naive infected animals’ lung, trachea, and nasal turbinates. Download Table S4, DOCX file, 0.05 MB. Copyright © 2023 Plunkard et al. 2023 Plunkard et al. https://creativecommons.org/licenses/by/4.0/ This content is distributed under the terms of the Creative Commons Attribution 4.0 International license.

Significant differences in mean viral titers were observed between variants for all three tissue types, with Omicron-infected animals displaying consistently lower tissue titers than other groups. In lung tissues, the most prominent difference was noted at 4 dpi, with the Omicron group having significantly lower lung titers than all other groups, while the Beta and Gamma groups had significantly higher titers than all groups except the Delta group (Fig. 4A). At 2 dpi, this trend was still appreciable and Omicron-infected animals had significantly lower titers than animals infected with other variants except A.2.5, B.1.1.207, and Epsilon, which were not significantly different (Fig. 4A; Table S4). Beta-infected animals had the highest overall lung titer and, along with Gamma-infected animals, had significantly higher titers than those infected with A.2.5, B.1.1.207, Epsilon, and Omicron. Delta-infected animals had comparable lung titers to those infected with A.2.5, A.3, B.1.1.207, Epsilon, and Gamma, while being significantly lower than Beta-infected animals and higher than Omicron-infected animals. Delta was the only variant with sex differences in lung titers at 2 dpi, with males having significantly higher mean titers than females (Fig. 4A). At 4 dpi, the only sex difference appreciated was B.1.1.207-infected males having significantly higher titers than females (Fig. 4A). Altogether, infectious viral titers in lung tissues were higher in Beta-infected animals and lower in Omicron-infected animals. These results correlate well with histopathology findings, supporting an early infection followed by a reparative phase for all reinfection groups (Fig. 3G).

In nasal turbinates at 2 dpi, Omicron-infected animals had significantly lower viral titers than those infected with A.3, Beta, and Gamma (Fig. 4B). Although the A.3, Beta, and Gamma groups trended toward having higher nasal turbinate titers at this time point, statistical tests suggested no significance. A.2.5-infected animals displayed sex differences in nasal turbinate titers at 2 dpi, with males having higher viral titer than females (Fig. 4B). At 4 dpi, Omicron-infected animals continued to have the significantly lowest nasal turbinate viral titer. Gamma-infected animals had the highest, with significantly higher titers than B.1.1.207-, Epsilon-, and Omicron-infected animals. At 7 dpi, low nasal turbinate titers were detectable for a few animals from multiple variant groups, with the exception of the Gamma group, which had detectable virus in 7 out of 8 hamsters’ nasal turbinates (Fig. 4B). Within the Gamma-infected animals at this time point, males demonstrated a significantly higher nasal turbinate titer than females.

At 2 dpi, Omicron-infected animals had the significantly lowest tracheal viral titer, while Delta-infected animals exhibited the highest (Fig. 4C). Tracheal tissue viral titers at 4 dpi had more variability between variants than other tissue types, but these differences were not statistically significant. There were no sex differences in tracheal titers at all time points.

### Reinfection.

**FIG 5:**
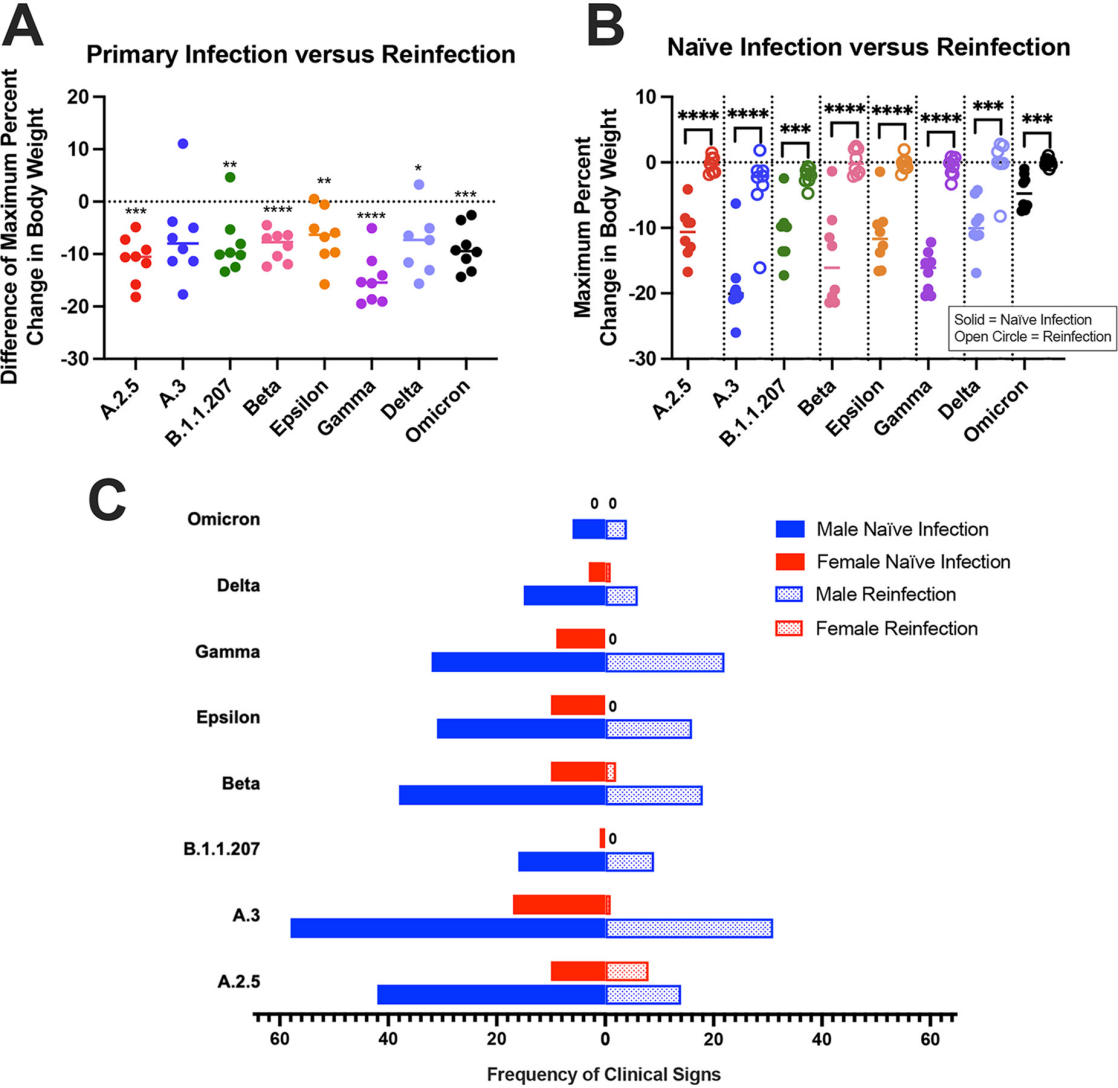
Clinical signs following reinfection. (A and B) Maximum percentage of body weight change over 7 dpi. (A) The difference in each hamster’s weight change between primary infection with variant A and reinfection with another variant was compared using paired *t* test, with almost all animals showing significantly less weight loss after reinfection. (B) Compared using two-sample *t* tests, naive hamsters infected with a variant lost significantly more weight than their counterparts that were reinfected with a variant after previous infection with variant A. (C) The frequency of clinical signs (hunched posture, orbital tightening, and rough hair coat) is shown for naive male and female hamsters at 7 dpi as well as male and female hamsters at reinfection at 7 dpi. Clinical signs were observed less in reinfected male hamsters versus naive male hamsters except for Omicron- and Delta-infected animals. A.3- and Gamma-reinfected females had significantly less clinical signs than their naive counterparts (*, *P* < 0.05; **, *P* < 0.01; ***, *P* < 0.001).

**(i) Clinical outcomes.** To investigate if previous SARS-CoV-2 infection ameliorates the frequency of clinical signs observed after reinfection, hamsters were inoculated with variant A and then a different variant 28 days later. After the first infection, hamsters lost up to 24% BW over the first 6 dpi and then began gaining weight around 7 dpi. They continued to gain BW until reinfection, after which their weights plateaued or slightly decreased immediately following inoculation, before increasing again, typically around 2 or 3 dpi. Figure S2A to H compare these trends in percentage of BW change after reinfection to that seen with naive infected animals for each variant. Figure 5A (see Table S2 for *P*-values) displays the difference in BW change for each hamster between its initial infection and reinfection, with significantly less weight loss seen after reinfection, except for animals infected with variant A.3 at 28 dpi, which showed no significant difference. The nonsignificance for the A.3-reinfected hamsters may be attributed to one hamster that exhibited a weight loss pattern similar to that of a naive infected animal. The mock-inoculated group lost significantly more BW following infection at 28 dpi than other groups. The mean percentage of BW change after reinfection was also significantly different from that of naive animals infected with the same variant, with naive animals losing more BW (Fig. 5B, see Table S2 for *P*-values). There were no sex differences in BW change in these groups following reinfection.

The presence and frequency of clinical signs were recorded over 7 dpi for both infections as described above. Male hamsters displayed more frequent clinical signs (rough hair coats, orbital tightening, and hunched posture) than females after both initial infections and reinfections (Fig. 5C). Significantly fewer clinical signs were observed after reinfection for males infected with A.2.5, B.1.1.207, Delta, and Omicron and females infected with Gamma. Almost all male groups displayed fewer clinical signs following reinfection than naive males infected with the same variant, except for those infected with B.1.1.207, Delta, and Omicron, which had a low frequency of clinical signs for both infections (Fig. 5C). Females previously infected with A.3 and Gamma also had significantly fewer clinical signs than their naïvely infected counterparts.

To evaluate if the initial variant influences reinfection outcomes, separate cohorts of animals were reinfected with Omicron 28 days after initial infection with Delta or Mu. These hamsters exhibited similar weight change trends to hamsters infected with variant A before Omicron reinfection (Fig. S3A and B). There were no significant differences in weight change between reinfected hamsters based on the variant they were initially infected with; however, all three groups of hamsters reinfected with Omicron had significantly less BW change than naive Omicron-infected hamsters. The frequency of clinical signs between reinfection groups was not significantly different.

10.1128/mbio.00078-23.3 FIG S3 Percentage of change in body weight after naive Omicron infection and Omicron reinfection. (A) Using a one-way ANOVA, the maximum percentages of change in body weight over 7 dpi were compared between naive hamsters infected with Omicron and hamsters reinfected with Omicron 28 days after initial infection with variant A, Mu, or Delta. There were no significant differences between reinfection groups, but all three reinfection groups had significantly less change in percentage of body weight than naive Omicron-infected hamsters (*P* < 0.05). (B) The percentage of change in body weight is shown over 7 dpi with Omicron. Naive hamsters infected with Omicron lost weight, while reinfected hamsters all gained weight on average regardless of their initial infection type. Download FIG S3, TIF file, 7.5 MB. Copyright © 2023 Plunkard et al. 2023 Plunkard et al. https://creativecommons.org/licenses/by/4.0/ This content is distributed under the terms of the Creative Commons Attribution 4.0 International license.

**FIG 6:**
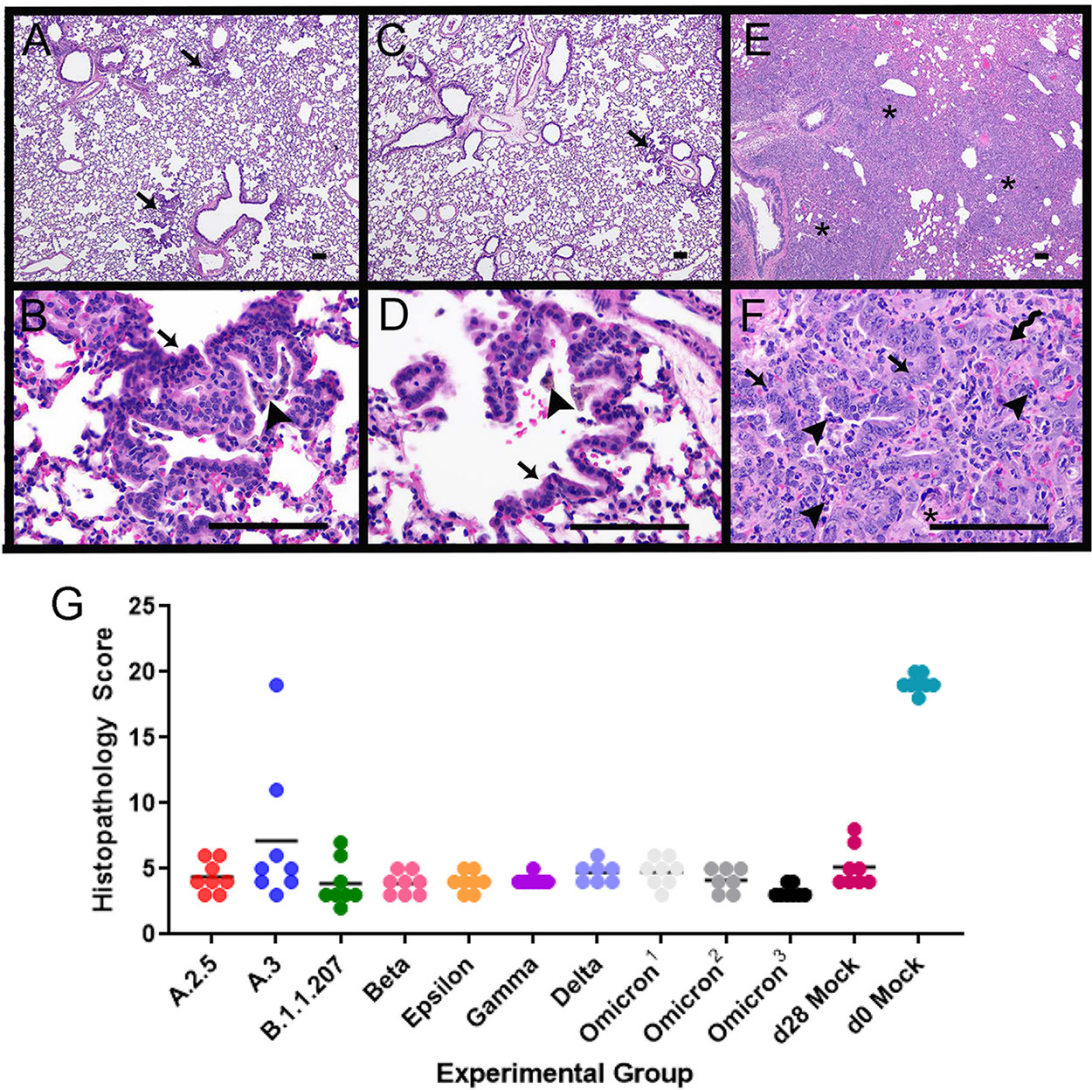
Histopathology of the lungs following reinfection. (A and B) Representative images from reinfection with the Delta variant. (A) Low magnification showing largely unremarkable lung with few foci of residual type II pneumocyte hyperplasia (arrows). (B) Higher magnification showing type II pneumocyte hyperplasia (arrow) and clusters of intra-alveolar clusters of pigmented macrophages (arrowhead). (C and D) Representative images from mock reinfection. (C) Low magnification showing largely unremarkable lung with foci of residual type II pneumocyte hyperplasia (arrow); (D) higher-magnification view of type II pneumocyte hyperplasia (arrow) and intra-alveolar pigmented macrophage (arrowhead). (E and F) Representative images from day 0 mock infection with variant reinfection. (E) Low-magnification view indicating widespread consolidation of the alveolar spaces (*); (F) higher magnification showing type II pneumocyte hyperplasia (arrows), intra-alveolar inflammatory cells, including neutrophils and macrophages (arrowheads), organizing fibrin within alveoli (*), and multinucleated epithelial cells (squiggle arrow). All scale bars represent 100 μm. (G) Histopathology scores of reinfection groups. Animals were intranasally infected with the A variant at day 0, infected with an additional variant at 28 dpi, and euthanized at 35 dpi. “Omicron^1^” represents initial infection with Delta and reinfection with Omicron. “Omicron^2^” represents initial infection with Mu and reinfection with Omicron. “Omicron^3^” represents initial infection with A and reinfection with Omicron. “d28 mock” indicates the animals were infected with the A variant at day 0 and mock inoculated with medium only at 28 dpi. “d0 mock” indicates the animals were mock inoculated with medium only at day 0 and inoculated with the A variant at 28 dpi. Please see Table S3 for a complete list of *P* values from multiple comparisons and one-way ANOVA. Secondary variant infection at 28 dpi produced similar histopathology scores. The d0 mock histopathology scores were statistically significantly higher (*P* < 0.0001 for multiple comparisons and one-way ANOVA) than all other groups. Variant A.3 had 2 animals with high histopathology scores, but scores were otherwise similar to all other groups.

**(ii) Histopathology.** Histopathology was performed on lung tissues at 7 dpi following reinfection (35 dpi initial infection) (Fig. 6). Animals had largely unaffected lungs 7 days after reinfection. In all variant groups, there were clusters of regular, cuboidal type II pneumocyte hyperplasia admixed with pigmented macrophages. Less consistent features across variants include alveolar hemorrhage and edema and perivascular lymphocytic aggregates.

10.1128/mbio.00078-23.5 TABLE S2 Results of multiple comparisons and one-way ANOVA of maximum percentage of body weight change for naive infected animals at 7 dpi, unpaired *t* test of maximum percentage of body weight change, and clinical sign frequency for naive infected animals at 7 dpi versus reinfected animals at 7 dpi, and paired *t* test of maximum percentage of body weight change for initial infection 7 dpi versus variant reinfection 7 dpi. Download Table S2, DOCX file, 0.04 MB. Copyright © 2023 Plunkard et al. 2023 Plunkard et al. https://creativecommons.org/licenses/by/4.0/ This content is distributed under the terms of the Creative Commons Attribution 4.0 International license.

10.1128/mbio.00078-23.6 TABLE S3 Results of multiple comparisons and one-way ANOVA of histology scoring for naive infected animals at 2, 4, and 7 dpi and reinfected animals at 7 dpi. Download Table S3, DOCX file, 0.04 MB. Copyright © 2023 Plunkard et al. 2023 Plunkard et al. https://creativecommons.org/licenses/by/4.0/ This content is distributed under the terms of the Creative Commons Attribution 4.0 International license.

In animals mock inoculated at day 28, there were clusters of type II pneumocyte hyperplasia with aggregates of pigmented macrophages, similar to hamsters that underwent two infections. In animals that were mock inoculated at day 0 and inoculated at day 28 with variant A, there was robust atypical type II pneumocyte hyperplasia, as described previously in the 7-dpi pathogenesis groups, along with abundant intra-alveolar and intraseptal macrophages. The day 0 mock-inoculated animals had statistically higher histopathology scores than all other groups. Animals infected with variant A.3 had statistically higher histopathology scores than the other groups, but this was largely attributed to one animal with widespread atypical type II pneumocyte hyperplasia and alveolar infiltrates as described for 7-dpi pathogenesis animals.

Tissue viral titer analyses were not performed for these groups as animals were euthanized at 7 days after reinfection, and low tissue viral loads at 7 dpi from this study and others have been reported (6, 26).

## DISCUSSION

SARS-CoV-2 variants have emerged with mutations affecting viral angiotensin-converting enzyme 2 (ACE2) binding affinity, replication, transmission, antibody recognition, and clinical disease (1, 27). Validation of animal models that accurately demonstrate disease caused by these variants is important for studying variant pathogenesis and assessing efficacy of therapies and vaccines. Here, Syrian hamsters infected with SARS-CoV-2 variants displayed clinical outcomes, lung pathology, and tissue viral titers, as previously described (28–30), with some differences observed between variants and between sexes within variant groups.

BW loss was a reliable clinical indicator of SARS-CoV-2 infection in this study. It was observed across variants regardless of sex, with naive hamsters losing weight over the first 6 dpi before beginning to recover on 7 dpi. This is consistent with previous studies reporting around 10 to 14% BW loss (7, 25, 26, 28–32) and similar patterns of recovery (6, 30). While all groups followed this pattern, some variant groups exhibited significant BW differences. A.3-infected animals demonstrated the most significant BW loss, particularly in males. This was an unexpected outcome as A.3 is genetically similar to the originally circulating virus, with minimal mutations, and the groups that exhibited relatively less weight loss were infected with variants possessing multiple mutations known to affect viral fusogenicity and infectivity (1, 27, 33, 34). Previous studies with a similar lineage did not report this severity of BW loss despite similar lung pathology (6, 26). We postulate that these differences could be due to variations in inoculation procedures or viral preparation. Despite the increased severity of clinical disease, A.3-infected animals had similar lung histopathology scores and tissue viral titers to most other groups. While this variant is no longer dominantly circulating among humans, our findings are useful as they highlight potential differences in SARS-CoV-2 infection between hamsters and humans.

The groups with the least BW loss were infected with Omicron, which is consistent with previous reports of Omicron clinical presentations in hamsters and human patients (35–40). In one study comparing Delta and Omicron infection in Syrian hamsters, Omicron caused less BW loss and lower viral loads in throat swabs and nasal wash samples (41). Due to its dominance in the population, Omicron is a priority for therapeutic and preventative research, particularly as it continues to evolve into multiple sublineages. In this study, we evaluated the early sublineage BA.1, referred to as Omicron throughout this article.

The etiology of BW loss in infected hamsters is not entirely understood, but it likely recapitulates the condition in human patients. Weight loss and clinical cachexia (muscle wasting from chronic disease) in people can be attributed to factors like loss of appetite and taste, anosmia, fever and inflammation, and metabolic imbalances (42, 43). While we did not evaluate appetite, taste, or smell, anosmia in infected hamsters has been reported to occur at 2 to 5 dpi (44, 45), which correlated with the period of maximal weight change (6, 30, 44, 45). Meanwhile, there is limited description on SAR-CoV-2 effects on hamster core body temperatures, but one study reported no changes following infection with an early variant (46). We postulate that the weight loss in hamsters is associated with inappetence, which could be a result of anosmia as correlated with the histological damage within the olfactory epithelium (44, 47). Additional studies examining the relationship of weight loss with these factors may elucidate its etiology.

The other clinical signs observed across variant groups were consistent with previous studies (28, 30); however, respiratory signs like rapid breathing have also been reported (29). As with our study, the majority of the literature describes minimal (6) to no (28) respiratory signs (25, 30). Such clinical severity differences could be attributed to factors like viral inoculation dose, with higher doses resulting in increased morbidity (29).

The influence of sex on clinical sign frequency in our animals mirrors the increased COVID-19 morbidity documented in male human patients (48–52). Male hamsters displayed more frequent clinical signs than females, which is also consistent with previous reports of increased morbidity in male hamsters (26, 51, 53). Moreover, intervariant differences in clinical sign frequencies were observed in male hamsters, suggesting they may be more sensitive to phenotypic effects of SARS-CoV-2 infection than females, both in the context of the initial infection and after reinfection. Of note, despite differences in the overall clinical sign frequencies, there were no sex differences associated with BW change up to 7 dpi for all groups, which is consistent with previous reports (26, 29, 51); however, some studies of longer duration noted that male hamsters regained less weight than females from 8 to 28 dpi (26, 51). Sex differences emphasize the importance of accounting for sex in SARS-CoV-2 research.

Overall, histopathological features were similar among all variants and consistent with previous literature (6, 7, 22, 25, 32, 54–56). Inflammatory lesions present at 2 dpi supported acute damage, which then progressed in severity by 4 dpi. In some animals, evidence of repair, such as type II pneumocyte hyperplasia, was already present by 4 dpi, but there was no obvious correlation to sex or variant with these repairs. At 7 dpi, lesions were consistent with further progression into the reparative phase, as characterized by extensive type II pneumocyte hyperplasia. In most variants, lesions were more extensive at 7 dpi; however, there were more features present at 4 dpi. As a result, two variants (B.1.1.207 and Beta) had higher histopathology scores at 4 dpi than 7 dpi. The histopathologic progression is consistent with the weight trends observed, with an immediate response to infection and gradual recovery toward 7 dpi. In people, respiratory lesions are primarily characterized by imaging, such as computed tomography (CT) (57, 58). The sensitivity of CT for diagnosing infection in humans increases significantly when symptom duration is longer than 48 h, after which increased lung consolidation and ground glass opacities are observed (58). CT has been used to evaluate SARS-CoV-2 in hamsters, with similar findings to human patients (7), and offers a viable option for evaluating lung pathology over time in this model (26, 30).

Omicron-infected animals had lower overall histopathology scores than most variants despite similar lesions observed (Fig. S1), likely due to lower percentages of affected tissue, fewer perivascular lymphocytes, and fewer atypical or multinucleated bronchial epithelial cells. Our findings differed slightly from previous reports that used lower inoculation doses, where hamsters demonstrated milder pneumonia when infected with Omicron than when infected with the Delta variant (35, 38). These studies found that Omicron-infected animals had milder features of pneumonia, including multiple small foci of inflammatory cells in the alveoli and peribronchial areas observed only at 6 dpi, with no changes noted at 3 dpi (38), or decreased areas of type II pneumocyte hyperplasia at 5 and 7 dpi (35).

All variant groups displayed a quick decrease in infectious virus load in respiratory tissues from 2 dpi to 4 dpi, and by 7 dpi, there were low to no detectable infectious viral particles. This pattern is similar to previous reports for hamsters infected with a clade A variant (6, 26, 29). The viral titer levels at 7 dpi coincide with the peak BW loss at 6 dpi and then recovery observed in this study and others (6). However, higher inoculation doses could result to consistently higher viral titer loads at 2 and 4 dpi, as previously reported (29). In our study, the high titer levels in the nasal turbinates and lungs may be attributed to the nasal cavity inoculation and more effective viral replication at 37°C in the lower respiratory track, respectively.

There was a reduction in clinical phenotype and lung pathology upon SARS-CoV-2 reinfection. Previously infected hamsters had significantly less BW loss than their naively infected counterparts, and the majority of groups had less frequent other clinical signs. Additionally, in 79/80 animals that were reinfected, lesions at 35 dpi (i.e., 7 days post-reinfection) were identical to those of the control group that received only one infection with variant A on day 0 and were mock inoculated at 28 dpi. These changes included residual type II pneumocyte hyperplasia and small foci of perivascular and intra-alveolar inflammatory cells, which were consistent with chronic, rather than acute, change. In contrast, animals mock inoculated at day 0 and inoculated at 28 dpi displayed changes more like those observed in 7-dpi animals. One animal in the reinfection cohort demonstrated findings similar to those found in animals euthanized at 7 dpi. Serology performed at 28 dpi on this hamster revealed no neutralizing antibodies (neutralizing antibody titer of <1:20 against the SARS-CoV-2 vaccine strain), indicating that there was likely a failure of primary inoculation. Protective antibody responses of variable durations are described in human cases, both from previous infection and vaccines (59–61). Overall, our findings indicate that some degree of protection against reinfection is generated during primary infection.

A.3- and Omicron-infected groups demonstrated clear phenotypic differences from other groups, supporting the recommendation that all variants should be characterized in the hamster model. Despite differences in reported transmissibility, viral replication, and clinical outcomes in humans and *ex vivo* studies (1, 2), the other variants analyzed were phenotypically similar. This suggests that although there are benefits as an animal model overall, the hamster model may not be specific enough to differentiate minor differences between certain variants. Despite this, preliminary studies on emerging variants using the model are still valuable for characterization before further investigation is conducted.

While we evaluated the effect of sex on different variants, there are other factors that can influence SARS-CoV-2 infection. Age has been shown to significantly affect disease outcomes in people (62–64) and in hamsters (22, 65). An additional limitation of our study is its duration; longer studies are needed to investigate effects of long COVID and the longevity of the protection from reinfection. Furthermore, evaluation of the hamster antibody response to different variants is important, as hamsters continue to be used for vaccine and monoclonal antibody (Mab) treatment research (66–68).

Emergence of new variants creates a continued need for SARS-CoV-2 research. Our study findings indicate that Syrian hamsters provide a reliable and consistent animal model for studying SARS-CoV-2 variant infections and reinfections, emphasizing its significance for characterizing disease and investigating effective treatments and vaccines.

## MATERIALS AND METHODS

### Virus preparation.

Virus preparation was performed as previously described by Mulka et al. (6). Briefly,Vero-E6-TMPRSS2 cells (from the Japan Institute of Infectious Diseases) were cultured in Dulbecco’s modified Eagle’s medium (DMEM) supplemented with 10% fetal bovine serum (FBS), 1 mM glutamine, 1 mM sodium pyruvate, 100 U/mL penicillin, and 100 mg/mL streptomycin. SARS-CoV-2 viruses from patient nasal swab samples collected at Johns Hopkins Hospital were used to infect Vero-E6-TMPRSS2 cells to generate virus stocks. Infection was done at a multiplicity of infection (MOI) of 0.01 50% tissue culture infective dose (TCID_50_). Infected-cell supernatant was collected when 75% of cells had observable cytopathic effect (CPE) around 96 h postinfection. The supernatant was centrifuged at 400 × *g* for 10 min and then aliquoted into 500 μL and stored at −70°C. The infectious titer of virus stock was measured by TCID_50_ assay. Virus stocks were 10-fold serially diluted in DMEM supplemented with 2.5% FBS, 1 mM glutamine, 1 mM sodium pyruvate, 100 U/mL penicillin, and 100 mg/mL streptomycin. Stock dilutions were transferred in sextuplicate into the 96-well plates confluent with Vero-E6-TMPRSS2 cells, incubated at 37°C for 6 days, and then fixed with 4% formaldehyde and stained with naphthol blue black solution for visualization. The infectious virus titers in TCID_50_ per milliliter were determined by the Reed and Muench method.

### Animals.

Male and female Syrian hamsters (6 to 8 weeks old) (Envigo, Haslett, MI) were singly housed in negative-pressure individually ventilated cages (PNC) (Allentown, Inc., Allentown, NJ). Animals were provided with corncob bedding (Envigo, Madison, WI), nesting material (Enviro-dri; Shepherd Specialty Papers, Amherst, MA), standard rodent chow (2018SX; Teklad, Envigo, Madison, WI) and RO-DI water through an automated watering system (Edstrom, Avidity Science, Waterford, WI). All animal procedures were approved by the Johns Hopkins Institutional Animal Care and Use Committee and conducted in an AAALAC-accredited facility.

**(i) Inoculation, clinical evaluation, and euthanasia.** Hamsters were sedated with ketamine-xylazine for inoculation with 10^5^ TCID_50_ of a SARS-COV-2 variant (Table 1) in 100 μL DMEM (50 μL/naris) or mock inoculated with 100 μL DMEM. Animals were weighed and clinically evaluated blind by an observer for the presence or absence of clinical signs (J.P.). At euthanasia, hamsters were anesthetized using isoflurane for cardiac puncture and tissue harvest.

**(ii) Naive SARS-COV-2 variant infection.** Hamsters (4/sex/endpoint/variant) were inoculated with a SARS-COV-2 variant. Daily observations and weighing were performed until euthanasia at 2, 4, and 7 dpi (Fig. 1A).

**(iii) Variant reinfection.** For primary infection, hamsters (4/sex/variant) were inoculated with 10^5^ TCID_50_ of SARS-CoV-2 130 USA-WA1/2020. Negative-control animals (4/sex) were mock inoculated. Twenty-eight days after induction of primary infection, experimental groups were inoculated with 10^5^ TCID_50_ of a variant (Table 1) and the initially mock-infected animals were inoculated with 10^5^ TCID_50_ of SARS-CoV-2 130 USA-WA1/2020. Animals were reinfected at 28 dpi because by this time, all animals had returned to their expected weights, showed no detrimental effects of the initial infection, and had reached peak levels of serum SARS-CoV-2 antibodies based on our previous experiments (6, 26). Periodic clinical assessment was performed until euthanasia at 35 dpi (Fig. 1B).

**(iv) Delta versus Mu effect on Omicron infection.** Hamsters (4/sex/variant) were inoculated with 10^5^ TCID_50_ of SARS-CoV-2/USA/MD-HP06587-PIDGNNWCBG/2021 or 10^5^ TCID_50_ of SARS-CoV-2/USA/MD-HP05660/2021. At 28 dpi, hamsters were inoculated again with 10^5^ TCID_50_ of SARS-CoV-2/USA/MD-HP20874-PIDUYWZOWA/2021. Clinical assessment and euthanasia were performed following the time points for the reinfection study described above.

### Histopathological analyses.

Histopathological analysis of lung tissues on hematoxylin and eosin (H&E) slides was performed blind by a veterinary pathologist (K.M.) using a scoring system shown in Table S1 in the supplemental material (24, 69, 70). Briefly, the parameters evaluated for their presence or absence include necrosis of bronchiolar epithelial cells (BECs), cellular debris in bronchi and bronchioles, cellular debris in alveoli, intra-alveolar fibrin, alveolar hemorrhage, alveolar edema, perivascular or interstitial edema, vasculitis, plump vascular endothelial cells, necrosuppurative bronchitis, hyperplasia of BEC, hyperplasia of type II alveolar epithelial cells (AECs), multinucleated or atypical BECs, and multinucleated or atypical AECs. Factors evaluated on a scale from 0 to 4 included percentage of lung affected and perivascular lymphocytes. Intra-alveolar neutrophils and macrophages were evaluated on a scale from 0 to 3.

### Tissue homogenization and viral titer analysis.

Animal tissue homogenization and infectious virus titration were done as previously described (6, 26). Briefly, tissue samples were transferred to Lysing Matrix D bead tubes on ice. DMEM supplemented with penicillin (100 U/mL) and streptomycin (100 mg/mL) was added to the tubes at a 10% (wt/vol) ratio. The samples were loaded in a FastPrep-24 benchtop bead beating system (MPBio) and homogenized for 40 s at 6.0 m/s, followed by centrifugation for 5 min at 10,000 × *g* at room temperature. Tubes were returned to ice, and supernatant was collected and stored at −70°C. The infectious virus titer in tissue homogenates was measure by TCID_50_ assay as described above.

### Statistical analysis.

Statistical analyses were performed using GraphPad Prism 9. Paired *t* tests were used to evaluate differences in BW and clinical signs in reinfection groups. One-sample *t* tests were used to evaluate sex differences within groups and clinical outcome differences between naive and reinfection. One-way analyses of variance (ANOVA) were used to evaluate differences between variant group BW, clinical signs, tissue titers, and histology scores. A significance of *P* < 0.05 was used for all tests.
