# Supplementary figures and images for: SARS-CoV-2 Variant Pathogenesis Following Primary Infection and Reinfection in Syrian Hamsters
Source: mBio. 2023 Apr 10;14(2):e00078-23. doi: 10.1128/mbio.00078-23 (PMC10128064; doi:10.1128/mbio.00078-23)

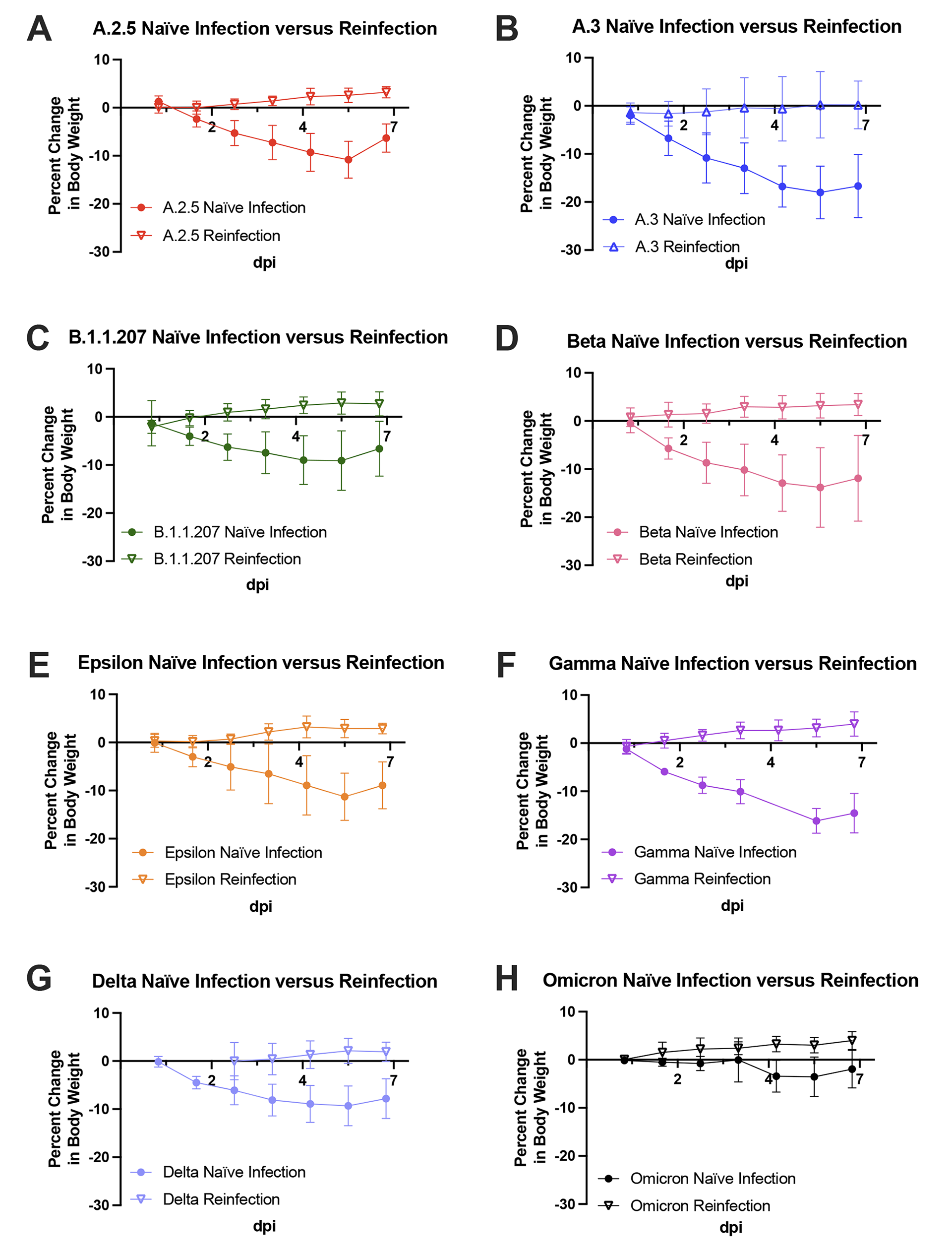

Supplement: FIG S2 [file mbio.00078-23-s0006.tif]

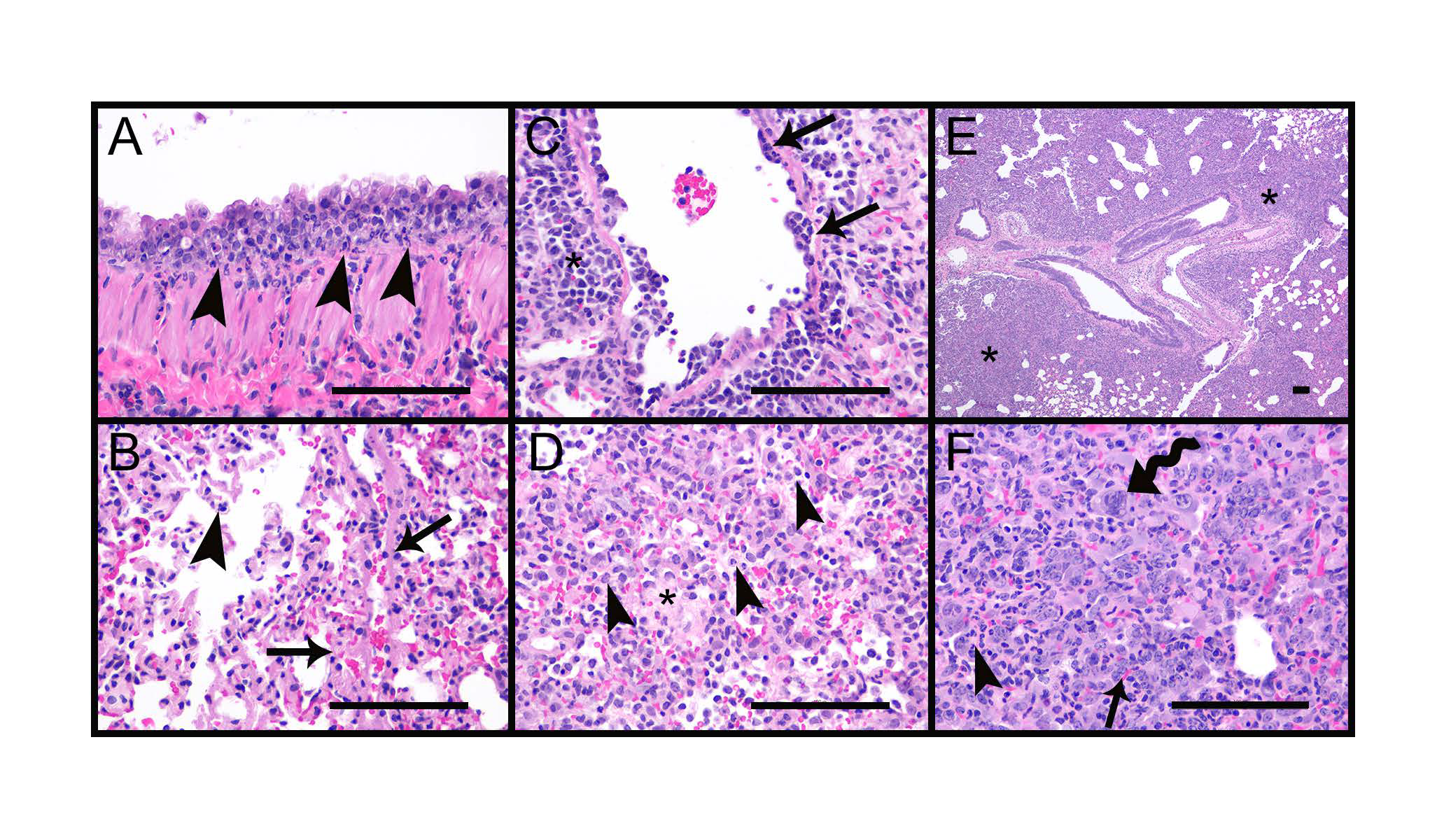

Supplement: FIG S1 [file mbio.00078-23-s0007.tif]

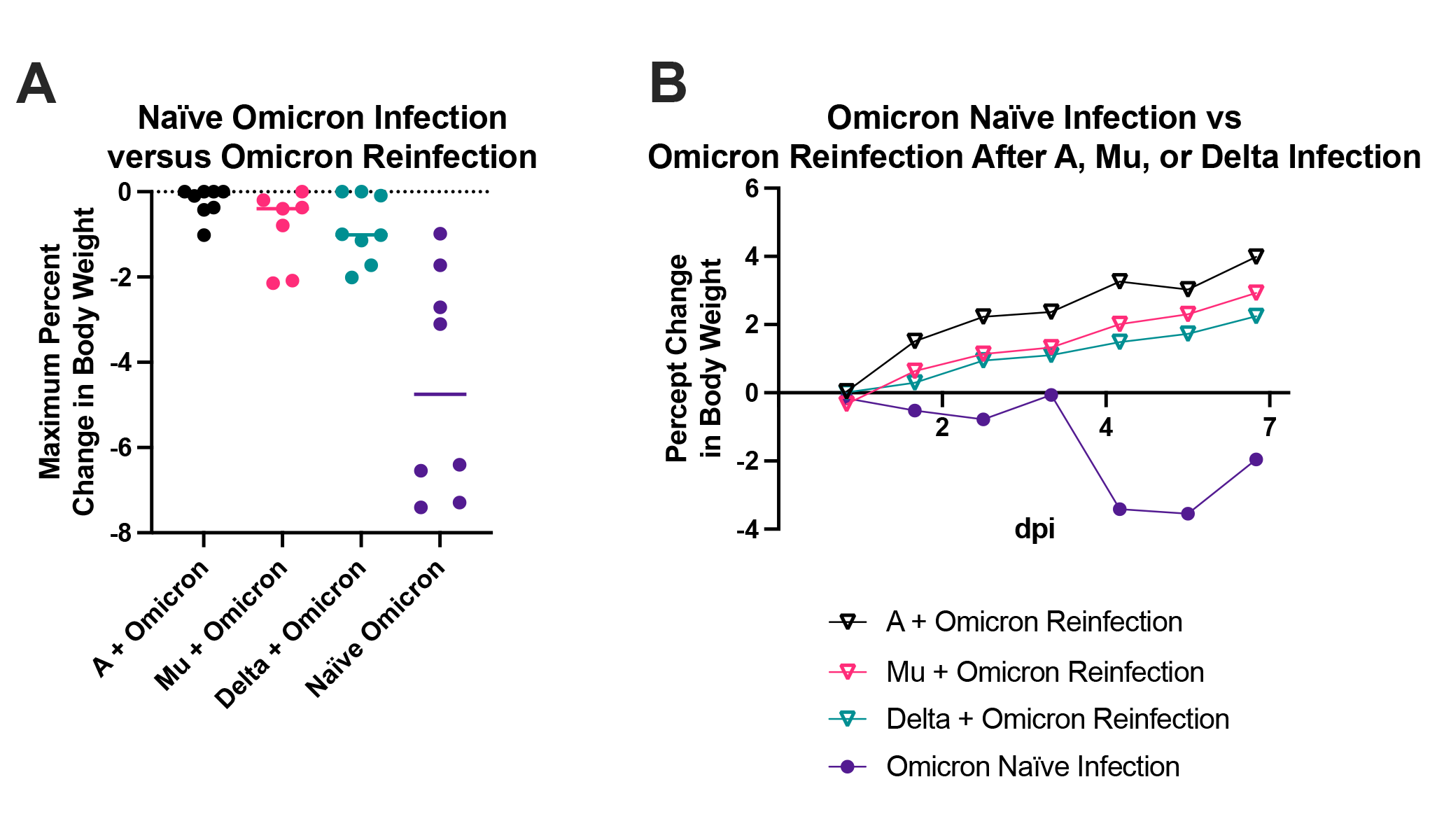

Supplement: FIG S3 [file mbio.00078-23-s0001.tif]
