# Supplementary material for: SARS-CoV-2 Variant Pathogenesis Following Primary Infection and Reinfection in Syrian Hamsters
Source: mBio. 2023 Apr 10;14(2):e00078-23. doi: 10.1128/mbio.00078-23 (PMC10128064; doi:10.1128/mbio.00078-23)
Supplement: TABLE S1 [file mbio.00078-23-s0002.docx]

**SUPPLEMENTAL TABLE 1** Histopathologic scoring system used by a blinded board-certified veterinary pathologist to analyze hamster lung tissue.

| **% Lung affected** | |
| --- | --- |
| None | 0 |
| <5% | 1 |
| <25% | 2 |
| <50% | 3 |
| >50% | 4 |
| **Distribution** | |
| Bronchial and peribronchial | 0/1 |
| Patchy throughout lungs | 0/1 |
| **Cell and Tissue Damage** | |
| Necrosis of BEC | 0/1 |
| Cellular Debris in bronchi/bronchioles | 0/1 |
| Necrosis of AEC | 0/1 |
| Cellular debris in alveoli | 0/1 |
| Intraalveolar fibrin | 0/1 |
| **Circulatory Changes and Vascular lesions** | |
| Alveolar hemorrhage | 0/1 |
| Alveolar edema | 0/1 |
| Perivascular/interstitial edema | 0/1 |
| Vasculitis/vascular endothelialitis | 0/1 |
| Plump vascular endothelial cells | 0/1 |

| **Inflammatory Patterns** | |
| --- | --- |
| Necrosuppurative bronchitis | 0/1 |
| Intraalveolar neutrophils and macrophages |  |
| None | 0 |
| increased inflammatory cells within alveoli, but not filling alveoli, septa clearly distinguished | 1 |
| inflammatory cells fill alveoli, septa clearly distinguished | 2 |
| inflammatory cells fill multiple adjacent alveoli, septa difficult to distinguish | 3 |
| Perivascular lymphocytes |  |
| None | 0 |
| 1 cell layer | 1 |
| 2-3 cell layers | 2 |
| 4-5 cell layers | 3 |
| >5 cell layers | 4 |
| **Regeneration and Repair** | |
| Hyperplasia of BEC | 0/1 |
| Hyperplasia of AEC-II | 0/1 |
| Multinucleated or Atypical bronchial Epithelial Cells | 0/1 |
| Multinucleated or Atypical alveolar Epithelial Cells | 0/1 |
