# Supplementary material for: SARS-CoV-2 Variant Pathogenesis Following Primary Infection and Reinfection in Syrian Hamsters
Source: mBio. 2023 Apr 10;14(2):e00078-23. doi: 10.1128/mbio.00078-23 (PMC10128064; doi:10.1128/mbio.00078-23)
Supplement: TABLE S4 [file mbio.00078-23-s0003.docx]

**SUPPLEMENTAL TABLE 4** Results of multiple comparisons one-way ANOVA of viral titer for 2, 4, and 7 dpi naïve infected animals’ lung, trachea, and nasal turbinates.

| **Lung viral titers 2dpi naïve infection** | | | | | | | | | |
| --- | --- | --- | --- | --- | --- | --- | --- | --- | --- |
|  | **A.2.5** | **A.3** | **B.1.1.207** | **Beta** | **Epsilon** | **Gamma** | **Delta** | **Omicron** | |
| **A.2.5** |  | ***0.0004 | >0.9999 | ****<0.0001 | 0.7891 | ****<0.0001 | 0.2977 | 0.9575 | |
| **A.3** |  |  | **0.0011 | 0.4592 | 0.0685 | 0.9991 | 0.3676 | ****<0.0001 | |
| **B.1.1.207** |  |  |  | ****<0.0001 | 0.9201 | ***0.0001 | 0.4801 | 0.8608 | |
| **Beta** |  |  |  |  | ****<0.0001 | 0.8286 | **0.001 | ****<0.0001 | |
| **Epsilon** |  |  |  |  |  | *0.0121 | 0.9943 | 0.1543 | |
| **Gamma** |  |  |  |  |  |  | 0.1114 | ****<0.0001 | |
| **Delta** |  |  |  |  |  |  |  | *0.0188 | |
| **Omicron** |  |  |  |  |  |  |  |  | |
| **Lung viral titers 4dpi naïve infection** | | | | | | | | | |
|  | **A.2.5** | **A.3** | **B.1.1.207** | **Beta** | **Epsilon** | **Gamma** | **Delta** | **Omicron** | |
| **A.2.5** |  | 0.9958 | 0.8623 | *0.0232 | >0.9999 | ****<0.0001 | 0.6555 | ****<0.0001 | |
| **A.3** |  |  | 0.998 | **0.0019 | 0.9966 | ****<0.0001 | 0.21 | ****<0.0001 | |
| **B.1.1.207** |  |  |  | ***0.0001 | 0.8721 | ****<0.0001 | *0.0415 | ****<0.0001 | |
| **Beta** |  |  |  |  | *0.0216 | 0.6263 | 0.7601 | ****<0.0001 | |
| **Epsilon** |  |  |  |  |  | ****<0.0001 | 0.6405 | ****<0.0001 | |
| **Gamma** |  |  |  |  |  |  | *0.0202 | ****<0.0001 | |
| **Delta** |  |  |  |  |  |  |  | ****<0.0001 | |
| **Omicron** |  |  |  |  |  |  |  |  | |
| **Lung viral titers 7dpi naïve infection** | | | | | | | | | |
|  | **A.2.5** | **A.3** | **B.1.1.207** | **Beta** | **Epsilon** | **Gamma** | **Delta** | **Omicron** | |
| **A.2.5** |  | >0.9999 | >0.9999 | >0.9999 | >0.9999 | >0.9999 | >0.9999 | >0.9999 | |
| **A.3** |  |  | >0.9999 | >0.9999 | >0.9999 | >0.9999 | >0.9999 | >0.9999 | |
| **B.1.1.207** |  |  |  | >0.9999 | >0.9999 | >0.9999 | >0.9999 | >0.9999 | |
| **Beta** |  |  |  |  | >0.9999 | >0.9999 | >0.9999 | >0.9999 | |
| **Epsilon** |  |  |  |  |  | >0.9999 | >0.9999 | >0.9999 | |
| **Gamma** |  |  |  |  |  |  | >0.9999 | >0.9999 | |
| **Delta** |  |  |  |  |  |  |  | >0.9999 | |
| **Omicron** |  |  |  |  |  |  |  |  | |
| **Trachea viral titers 2dpi naïve infection** | | | | | | | | |  |
|  | **A.2.5** | **A.3** | **B.1.1.207** | **Beta** | **Epsilon** | **Gamma** | **Delta** | **Omicron** |  |
| **A.2.5** |  | 0.0535 | 0.5398 | 0.7059 | >0.9999 | 0.8984 | ****<0.0001 | **0.0037 |  |
| **A.3** |  |  | ****<0.0001 | 0.8678 | 0.1515 | 0.6567 | *0.0112 | ****<0.0001 |  |
| **B.1.1.207** |  |  |  | *0.0101 | 0.2833 | *0.0326 | ****<0.0001 | 0.5128 |  |
| **Beta** |  |  |  |  | 0.9123 | >0.9999 | ****<0.0001 | ****<0.0001 |  |
| **Epsilon** |  |  |  |  |  | 0.9869 | ****<0.0001 | ***0.0008 |  |
| **Gamma** |  |  |  |  |  |  | ****<0.0001 | ****<0.0001 |  |
| **Delta** |  |  |  |  |  |  |  | ****<0.0001 |  |
| **Omicron** |  |  |  |  |  |  |  |  |  |

| **Trachea viral titers 4dpi naïve infection** | | | | | | | | |
| --- | --- | --- | --- | --- | --- | --- | --- | --- |
|  | **A.2.5** | **A.3** | **B.1.1.207** | **Beta** | **Epsilon** | **Gamma** | **Delta** | **Omicron** |
| **A.2.5** |  | >0.9999 | 0.1954 | 0.9987 | 0.4417 | >0.9999 | 0.8416 | 0.5052 |
| **A.3** |  |  | 0.2246 | 0.9994 | 0.4863 | >0.9999 | 0.8722 | 0.551 |
| **B.1.1.207** |  |  |  | 0.5499 | 0.9998 | 0.1799 | 0.9589 | 0.9994 |
| **Beta** |  |  |  |  | 0.8312 | 0.998 | 0.9919 | 0.8752 |
| **Epsilon** |  |  |  |  |  | 0.4167 | 0.9983 | >0.9999 |
| **Gamma** |  |  |  |  |  |  | 0.8224 | 0.4793 |
| **Delta** |  |  |  |  |  |  |  | 0.9994 |
| **Omicron** |  |  |  |  |  |  |  |  |
| **Trachea viral titers 7dpi naïve infection** | | | | | | | | |
|  | **A.2.5** | **A.3** | **B.1.1.207** | **Beta** | **Epsilon** | **Gamma** | **Delta** | **Omicron** |
| **A.2.5** |  | >0.9999 | >0.9999 | >0.9999 | >0.9999 | >0.9999 | >0.9999 | >0.9999 |
| **A.3** |  |  | >0.9999 | >0.9999 | >0.9999 | >0.9999 | >0.9999 | >0.9999 |
| **B.1.1.207** |  |  |  | >0.9999 | >0.9999 | >0.9999 | >0.9999 | >0.9999 |
| **Beta** |  |  |  |  | >0.9999 | >0.9999 | >0.9999 | >0.9999 |
| **Epsilon** |  |  |  |  |  | >0.9999 | >0.9999 | >0.9999 |
| **Gamma** |  |  |  |  |  |  | >0.9999 | >0.9999 |
| **Delta** |  |  |  |  |  |  |  | >0.9999 |
| **Omicron** |  |  |  |  |  |  |  |  |
| **Nasal Turbinates viral titers 2dpi naïve infection** | | | | | | | | |
|  | **A.2.5** | **A.3** | **B.1.1.207** | **Beta** | **Epsilon** | **Gamma** | **Delta** | **Omicron** |
| **A.2.5** |  | 0.2694 | >0.9999 | 0.2075 | 0.9616 | 0.2079 | 0.9954 | 0.2482 |
| **A.3** |  |  | 0.1613 | >0.9999 | 0.9033 | >0.9999 | 0.7431 | ***0.0001 |
| **B.1.1.207** |  |  |  | 0.119 | 0.8899 | 0.1193 | 0.9747 | 0.3862 |
| **Beta** |  |  |  |  | 0.8487 | >0.9999 | 0.6597 | ****<0.0001 |
| **Epsilon** |  |  |  |  |  | 0.8493 | >0.9999 | *0.0148 |
| **Gamma** |  |  |  |  |  |  | 0.6604 | ****<0.0001 |
| **Delta** |  |  |  |  |  |  |  | *0.0401 |
| **Omicron** |  |  |  |  |  |  |  |  |
| **Nasal Turbinates viral titers 4dpi naïve infection** | | | | | | | | |
|  | **A.2.5** | **A.3** | **B.1.1.207** | **Beta** | **Epsilon** | **Gamma** | **Delta** | **Omicron** |
| **A.2.5** |  | 0.5567 | 0.7599 | 0.9997 | 0.7113 | 0.3313 | 0.9231 | ***0.0002 |
| **A.3** |  |  | *0.0146 | 0.2606 | *0.0112 | >0.9999 | 0.9977 | ****<0.0001 |
| **B.1.1.207** |  |  |  | 0.9555 | >0.9999 | **0.0044 | 0.0997 | 0.0517 |
| **Beta** |  |  |  |  | 0.9361 | 0.1225 | 0.683 | **0.0013 |
| **Epsilon** |  |  |  |  |  | **0.0033 | 0.0814 | 0.0643 |
| **Gamma** |  |  |  |  |  |  | 0.9712 | ****<0.0001 |
| **Delta** |  |  |  |  |  |  |  | ****<0.0001 |
| **Omicron** |  |  |  |  |  |  |  |  |

| **Nasal Turbinates viral titers 7dpi naïve infection** | | | | | | | | |
| --- | --- | --- | --- | --- | --- | --- | --- | --- |
|  | **A.2.5** | **A.3** | **B.1.1.207** | **Beta** | **Epsilon** | **Gamma** | **Delta** | **Omicron** |
| **A.2.5** |  | 0.9485 | 0.9554 | 0.9916 | >0.9999 | 0.2087 | 0.9998 | >0.9999 |
| **A.3** |  |  | >0.9999 | >0.9999 | 0.9881 | 0.877 | 0.9972 | 0.9485 |
| **B.1.1.207** |  |  |  | >0.9999 | 0.9904 | 0.8639 | 0.9979 | 0.9554 |
| **Beta** |  |  |  |  | 0.9992 | 0.7112 | >0.9999 | 0.9916 |
| **Epsilon** |  |  |  |  |  | 0.3425 | >0.9999 | >0.9999 |
| **Gamma** |  |  |  |  |  |  | 0.4569 | 0.2087 |
| **Delta** |  |  |  |  |  |  |  | 0.9998 |
| **Omicron** |  |  |  |  |  |  |  |  |
