# Supplementary material for: SARS-CoV-2 Variant Pathogenesis Following Primary Infection and Reinfection in Syrian Hamsters
Source: mBio. 2023 Apr 10;14(2):e00078-23. doi: 10.1128/mbio.00078-23 (PMC10128064; doi:10.1128/mbio.00078-23)
Supplement: TABLE S2 [file mbio.00078-23-s0005.docx]

**SUPPLEMENTAL TABLE 2** Results of multiple comparisons one-way ANOVA of maximum percent body weight change for 7 dpi naïve infected animals, unpaired T-test of maximum percent body weight change and clinical sign frequency for 7 dpi naïve infected animals vs 7 dpi reinfected animals, and paired T-test of maximum percent body weight change for 7 dpi initial infection vs 7 dpi variant reinfection.

**Maximum percent body weight change for 7 dpi naïve infected animals**

|  | **A.2.5** | **A.3** | **B.1.1.207** | **Beta** | **Epsilon** | **Gamma** | **Delta** | **Omicron** |
| --- | --- | --- | --- | --- | --- | --- | --- | --- |
| **A.2.5** |  | 0.0224 | >0.9999 | 0.7275 | >0.9999 | 0.2157 | 0.9995 | 0.1504 |
| **A.3** |  |  | 0.0193 | 0.6134 | 0.0387 | 0.9799 | 0.0049 | <0.0001 |
| **B.1.1.207** |  |  |  | 0.6948 | >0.9999 | 0.1943 | 0.9998 | 0.1681 |
| **Beta** |  |  |  |  | 0.8383 | 0.9877 | 0.3973 | 0.0016 |
| **Epsilon** |  |  |  |  |  | 0.3117 | 0.9961 | 0.0958 |
| **Gamma** |  |  |  |  |  |  | 0.0694 | ****<0.0001 |
| **Delta** |  |  |  |  |  |  |  | 0.3879 |
| **Omicron** |  |  |  |  |  |  |  |  |

**Unpaired t-test maximum percent body weight change for 7 dpi naïve infected animals vs 7 dpi reinfected animals**

| **A.2.5** | ****<0.0001 |
| --- | --- |
| **A.3** | ****<0.0001 |
| **B.1.1.207** | ***0.0001 |
| **Beta** | ****<0.0001 |
| **Epsilon** | ****<0.0001 |
| **Gamma** | ****<0.0001 |
| **Delta** | ***0.0005 |
| **Omicron** | ***0.0003 |

**Unpaired t-test male clinical sign frequency for 7 dpi naïve infected animals vs 7 dpi reinfected animals**

| **A.2.5** | **0.0016 |
| --- | --- |
| **A.3** | *0.0148 |
| **B.1.1.207** | 0.3014 |
| **Beta** | *0.0278 |
| **Epsilon** | *0.0458 |
| **Gamma** | 0.0082 |
| **Delta** | 0.4699 |
| **Omicron** | 0.6704 |

**Unpaired t-test female clinical sign frequency for 7 dpi naïve infected animals vs 7 dpi reinfected animals**

| **A.2.5** | 0.7586 |
| --- | --- |
| **A.3** | **0.0093 |
| **B.1.1.207** | 0.3559 |
| **Beta** | 0.2666 |
| **Epsilon** | 0.2347 |
| **Gamma** | *0.0388 |
| **Delta** | 0.646 |
| **Omicron** | N/A all values same |

**Paired t-test maximum percent body weight change for 7 dpi initial infection vs 7 dpi variant reinfection**

| **A.2.5** | ***0.0002 |
| --- | --- |
| **A.3** | 0.0574 |
| **B.1.1.207** | **0.0052 |
| **Beta** | ****<0.0001 |
| **Epsilon** | 0.009 |
| **Gamma** | ****<0.0001 |
| **Delta** | *0.0151 |
| **Omicron** | ***0.0008 |

**Paired t-test male clinical sign frequency for 7 dpi initial infection vs 7 dpi variant reinfection**

| **A.2.5** | *0.0486 |
| --- | --- |
| **A.3** | 0.65 |
| **B.1.1.207** | *0.011 |
| **Beta** | 0.2452 |
| **Epsilon** | 0.0774 |
| **Gamma** | 0.093 |
| **Delta** | *0.0247 |
| **Omicron** | **0.0034 |

**Paired t-test female clinical sign frequency for 7 dpi initial infection vs 7 dpi variant reinfection**

| **A.2.5** | 0.3677 |
| --- | --- |
| **A.3** | >0.9999 |
| **B.1.1.207** | 0.1817 |
| **Beta** | 0.391 |
| **Epsilon** | 0.1328 |
| **Gamma** | *0.0305 |
| **Delta** | 0.8439 |
| **Omicron** | 0.2522 |

**Maximum percent body weight change for 7 dpi Omicron infected animals**

|  | **Naïve Omicron** | **A + Omicron** | **Mu + Omicron** | **Delta + Omicron** |
| --- | --- | --- | --- | --- |
| **Naïve Omicron** |  | ****<0.0001 | ***0.0003 | ***0.0002 |
| **A + Omicron** |  |  | 0.8517 | 0.8260 |
| **Mu + Omicron** |  |  |  | >0.9999 |
| **Delta + Omicron** |  |  |  |  |
