# Supplementary material for: SARS-CoV-2 Variant Pathogenesis Following Primary Infection and Reinfection in Syrian Hamsters
Source: mBio. 2023 Apr 10;14(2):e00078-23. doi: 10.1128/mbio.00078-23 (PMC10128064; doi:10.1128/mbio.00078-23)
Supplement: TABLE S3 [file mbio.00078-23-s0004.docx]

**SUPPLEMENTAL TABLE 3** Results of multiple comparisons one-way ANOVA of histology scoring for 2, 4, and 7 dpi naïve infected animals and 7 dpi reinfected animals.

**Lung histology scores for 2dpi naïve infected animals**

|  | **A.2.5** | **A.3** | **B.1.1.207** | **Beta** | **Epsilon** | **Gamma** | **Delta** | **Omicron** |
| --- | --- | --- | --- | --- | --- | --- | --- | --- |
| **A.2.5** |  | >0.99 | 0.81 | 0.77 | 0.89 | 0.87 | 0.99 | 0.07 |
| **A.3** |  |  | 0.9 | 0.87 | 0.8 | 0.77 | 0.99 | 0.04* |
| **B.1.1.207** |  |  |  | >0.99 | 0.13 | 0.11 | 0.71 | 0.0009*** |
| **Beta** |  |  |  |  | 0.11 | 0.08 | 0.65 | 0.0006*** |
| **Epsilon** |  |  |  |  |  | >0.99 | 0.95 | 0.74 |
| **Gamma** |  |  |  |  |  |  | 0.94 | 0.71 |
| **Delta** |  |  |  |  |  |  |  | 0.11 |

**Lung histology scores for 4dpi naïve infected animals**

|  | **A.2.5** | **A.3** | **B.1.1.207** | **Beta** | **Epsilon** | **Gamma** | **Delta** | **Omicron** |
| --- | --- | --- | --- | --- | --- | --- | --- | --- |
| **A.2.5** |  | >0.99 | >0.99 | 0.53 | 0.61 | 0.98 | 0.05 | 0.0005*** |
| **A.3** |  |  | 0.99 | 0.38 | 0.76 | 0.99 | 0.09 | 0.0011** |
| **B.1.1.207** |  |  |  | 0.76 | 0.38 | 0.88 | 0.02* | 0.0002*** |
| **Beta** |  |  |  |  | 0.0094** | 0.09 | 0.0001*** | <0.0001**** |
| **Epsilon** |  |  |  |  |  | 0.99 | 0.88 | 0.09 |
| **Gamma** |  |  |  |  |  |  | 0.38 | 0.01* |
| **Delta** |  |  |  |  |  |  |  | 0.73 |

**Lung histology scores for 7dpi naïve infected animals**

|  | **A.2.5** | **A.3** | **B.1.1.207** | **Beta** | **Epsilon** | **Gamma** | **Delta** | **Omicron** |
| --- | --- | --- | --- | --- | --- | --- | --- | --- |
| **A.2.5** |  | 0.99 | 0.0004*** | 0.44* | 0.98 | 0.0001*** | <0.0001**** | <0.0001**** |
| **A.3** |  |  | 0.0019** | 0.12 | 0.99 | 0.0004*** | <0.0001**** | <0.0001**** |
| **B.1.1.207** |  |  |  | 0.81 | 0.0071** | 0.99 | 0.19 | 0.68 |
| **Beta** |  |  |  |  | 0.29 | 0.54 | 0.0037** | 0.044* |
| **Epsilon** |  |  |  |  |  | 0.0019** | <0.0001**** | <0.0001**** |
| **Gamma** |  |  |  |  |  |  | 0.41 | 0.9 |
| **Delta** |  |  |  |  |  |  |  | 0.99 |

**Lung histology scores for reinfected animals 7dpi post reinfection**

|  | **A.2.5** | **A.3** | **B.1.1.207** | **Beta** | **Epsilon** | **Gamma** | **d0 Mock** | **Delta** | **d28 Mock** | **Delta+Omicron** | **Mu+Omicron** | **WA+Omicron** |
| --- | --- | --- | --- | --- | --- | --- | --- | --- | --- | --- | --- | --- |
| **A.2.5** |  | 0.14 | >0.99 | >0.99 | >0.99 | >0.99 | <0.0001**** | >0.99 | 0.99 | >0.99 | >0.99 | 0.99 |
| **A.3** |  |  | 0.03* | 0.03* | 0.048* | 0.069 | <0.0001**** | 0.34 | 0.58 | 0.31 | 0.096 | 0.0038** |
| **B.1.1.207** |  |  |  | >0.99 | >0.99 | >0.99 | <0.0001**** | 0.99 | 0.97 | 0.99 | >0.99 | >0.99 |
| **Beta** |  |  |  |  | >0.99 | >0.99 | <0.0001**** | 0.99 | 0.97 | 0.99 | >0.99 | >0.99 |
| **Epsilon** |  |  |  |  |  | >0.99 | <0.0001**** | 0.99 | 0.99 | 0.99 | >0.99 | 0.99 |
| **Gamma** |  |  |  |  |  |  | <0.0001**** | >0.99 | 0.99 | >0.99 | >0.99 | 0.99 |
| **d0 Mock** |  |  |  |  |  |  |  | <0.0001**** | <0.0001**** | <0.0001**** | <0.0001**** | <0.0001**** |
| **Delta** |  |  |  |  |  |  |  |  | >0.99 | >0.99 | >0.99 | 0.93 |
| **d28 Mock** |  |  |  |  |  |  |  |  |  | >0.99 | 0.99 | 0.67 |
| **Delta+Omicron** |  |  |  |  |  |  |  |  |  |  | >0.99 | 0.9 |
| **Mu+Omicron** |  |  |  |  |  |  |  |  |  |  |  | 0.99 |
